# A Straightforward Method for the Development of Positively Charged Gold Nanoparticle-Based Vectors for Effective siRNA Delivery

**DOI:** 10.3390/molecules28083318

**Published:** 2023-04-08

**Authors:** Tatiana N. Elizarova, Maxim L. Antopolsky, Denis O. Novichikhin, Artemiy M. Skirda, Alexey V. Orlov, Vera A. Bragina, Petr I. Nikitin

**Affiliations:** 1 Prokhorov General Physics Institute, Russian Academy of Sciences, 119991 Moscow, Russia; 2 National Research Nuclear University MEPhI (Moscow Engineering Physics Institute), 115409 Moscow, Russia

**Keywords:** siRNA delivery, cell-penetrating peptides, gold nanoparticles, secreted alkaline phosphatase

## Abstract

The therapeutic potential of short interfering RNA (siRNA) to treat many diseases that are incurable with traditional preparations is limited by the extensive metabolism of serum nucleases, low permeability through biological membrane barriers because of a negative charge, and endosomal trapping. Effective delivery vectors are required to overcome these challenges without causing unwanted side effects. Here, we present a relatively simple synthetic protocol to obtain positively charged gold nanoparticles (AuNPs) with narrow size distribution and the surface modified with Tat-related cell-penetrating peptide. The AuNPs were characterized using TEM and the localized surface plasmon resonance technique. The synthesized AuNPs showed low toxicity in experiments in vitro and were able to effectively form complexes with double-stranded siRNA. The obtained delivery vehicles were used for intracellular delivery of siRNA in an ARPE-19 cell line transfected with secreted embryonic alkaline phosphatase (SEAP). The delivered oligonucleotide remained intact and caused a significant knockdown effect on SEAP cell production. The developed material could be useful for delivery of negatively charged macromolecules, such as antisense oligonucleotides and various RNAs, particularly for retinal pigment epithelial cell drug delivery.

## 1. Introduction

Small interfering RNA (siRNA) has shown great promise as a tool for targeted gene silencing, making it a promising direction for the development of novel small-molecule-based therapeutics [1]. In 2018, the FDA inaugurated this revolutionary approach by approval of the first siRNA drug—patisiran [2]. At present, tens of siRNA-based drugs are in clinical trials for the treatment of various cancers, genetic disorders, and viral infections [3]. Despite their promise as therapeutic agents, the utilization of siRNAs is limited by their suboptimal pharmacokinetics. Their unfavorable characteristics include rapid degradation by serum nucleases and rapid elimination from the body by the kidneys [4]. Furthermore, siRNAs struggle to penetrate cell membranes due to their negative charge [5,6], and even when they do enter cells through endocytosis, they are often retained in endosomes [7]. Thus, an effective delivery vector is required to protect siRNAs from nuclease degradation, mediate their delivery, and release to target cells, without unwanted side effects or, alternatively, utilize non-endocytic pathways for direct cellular internalization of siRNA into the cytoplasm. 

Previous reports on improving siRNA delivery were focused on the chemical stabilization of siRNA [8,9]. Despite a certain degree of improvement, the use of naked siRNA was limited due to its poor cell membrane permeability; however, it was later shown that nanoparticle-mediated siRNA delivery was more beneficial in terms of siRNA protection from degradation and improved cellular uptake, as well as elevated efficacy [10]. Many of the existing delivery methods (e.g., lipids-based) provide enhanced endosomal escape [11] and also show significant safety concerns [12]. One way to circumvent these issues could be enhancing the nuclear localization of siRNA by means of cell-penetrating peptides (CPPs). Due to more effective internalization and facilitated nuclear localization, CPPs are deemed to be effective in reducing the effective siRNA dose, and, hence, any side effects.

Most CPPs are short cationic peptides (6–30 amino acid residues) known for facilitating membrane translocation and/or nuclear localization [13]. They exert membrane translocation via mechanisms that remain under investigation [13,14]. The cellular uptake and subsequent gene silencing efficiency of siRNA can be greatly improved by forming non-covalent complexes containing CPPs and siRNA [15,16,17,18], by covalently conjugating them [19,20,21] or by encapsulating siRNA within CPP-functionalized nanoparticles. Some encouraging results have also been seen in vitro [22,23,24] and in vivo [25,26] after complexing siRNA to endosomal escape-facilitating CPPs. The Tat48-60 peptide, derived from the Tat protein of the human immunodeficiency virus (HIV), is a highly promising CPP due to its ability to effectively traverse cell membranes and deliver attached agents into the nucleus of target cells [27]. It has been extensively studied as a delivery vector for biomolecules [28] and various nanoparticles [29,30,31,32,33,34,35,36].

Utilizing nanoparticles for siRNA delivery allows for its multimerization, and such delivery vectors can be designed in a way to provide multifunctionality and, hence, enhanced delivery. Gold nanoparticles (AuNPs) have become a popular choice for core nanoparticle scaffolds due to their ability to be easily detected and quantified, as well as because of their potential biocompatibility resulting from their chemical stability. These attributes have led to their extensive use in diagnostic and therapeutic areas of research [37,38,39,40,41,42]. In recent years, AuNPs have gained popularity as delivery vehicles for a variety of bioactive agents, including proteins and peptides [43], plasmid DNA [44,45], and antisense oligonucleotides [46,47]. One of the most popular approaches for biofunctionalizing AuNPs with siRNA is through the direct immobilization of thiolated siRNA onto the surface [31,48,49]. Another approach to obtain siRNA/AuNP complexes is by modifying the surface of nanoparticles with cysteamine [50,51], polyethyleneimine [52,53,54,55], or other cations [56], which imparts a positive charge to the surface, enabling the subsequent non-covalent sorption of siRNA.

Many such constructs employ a layer-by-layer approach to further coat the attached siRNA layer with PEI [29,57], charge reversal polyelectrolytes for endosome disruption [58], and/or hyaluronic acid for active targeting [59]. However, the addition of extra functionality as a CPP for better internalization is usually performed in a complex manner. For instance, while one siRNA strand was thiolated in order to coordinate with the gold surface, the second strand was capped with biotin used to form a biotin–streptavidin–biotin “sandwich” to display biotinylated Tat [31,60]. Moreover, the few examples of successful in vivo RNAi achieved with AuNPs were performed using highly complex systems consisting of five or more hierarchically assembled components [61,62].

To avoid a multi-component, multi-step, and multi-layer approach but to maintain multifunctionality, a new method was devised. Here, we describe a simple, reproducible one-step method for the synthesis of positively charged AuNPs coated with cysteamine and displaying a cationic CPP, a modification of the Tat48-60 sequence. This nanoconjugate demonstrates a good ability to complex molecules of nucleic acid nature and low cytotoxicity. The method was employed for the construction of siRNA/AuNP complexes and further transfection of ARPE-19 cells that produce the reporter molecule, secreted alkaline phosphatase (SEAP). Our results show that Tat-siRNA-modified AuNPs could serve as vehicles for gene therapy via intracellular delivery without the loss of biological activity. The developed material can be a useful tool in drug delivery of negatively charged macromolecules such as antisense oligonucleotides, different RNAs, etc.

## 2. Results

### 2.1. Synthesis of CPP-AuNPs and Their Characterization

**Scheme 1:**
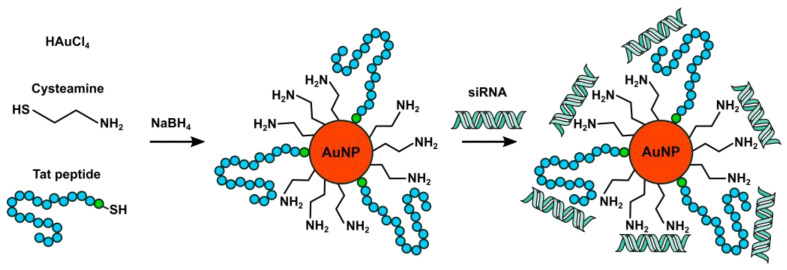
Preparation of peptide surface-modified AuNPs.

**Figure 1:**
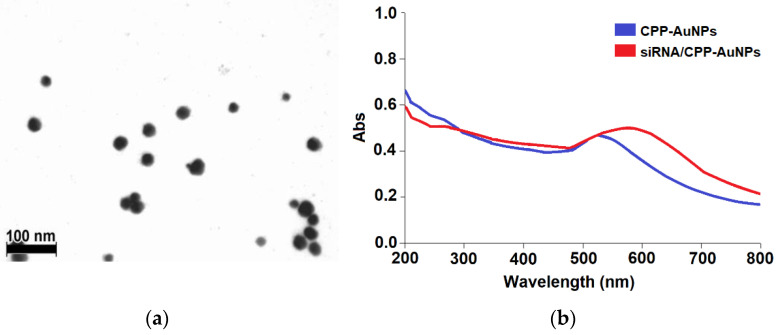
(**a**) TEM image of CPP-AuNPs and (**b**) absorption analysis with UV-Vis spectroscopy: CPP-AuNPs (blue line) and siRNA/CPP-AuNPs (red line).

The proposed method involves synthesizing gold clusters, which are stabilized by an alkanethiol monolayer in a two-phase aqueous-organic environment using the modified Brust–Schiffrin method [63]. It employs NaBH4 as the reducing agent, typically yielding ~30-nanometer cysteamine-coated cationic AuNPs [64]. The key concept of the modification lies in the simultaneous addition of cysteamine and the required peptides, as described in Scheme 1. As a result, it is possible to get mixed monolayers of cysteamine moieties and several CPPs on the nanoparticle surface. The shape and size of the particles were studied with TEM. The obtained CPP-AuNPs exhibited the characteristic features of gold nanospheres (Figure 1a) with a local plasmon resonance peak at 520 nm (Figure 1b), as confirmed by UV–Vis absorption spectroscopy analysis.

### 2.2. Investigation of siRNA/CPP-GNP Stability

**Figure 2:**
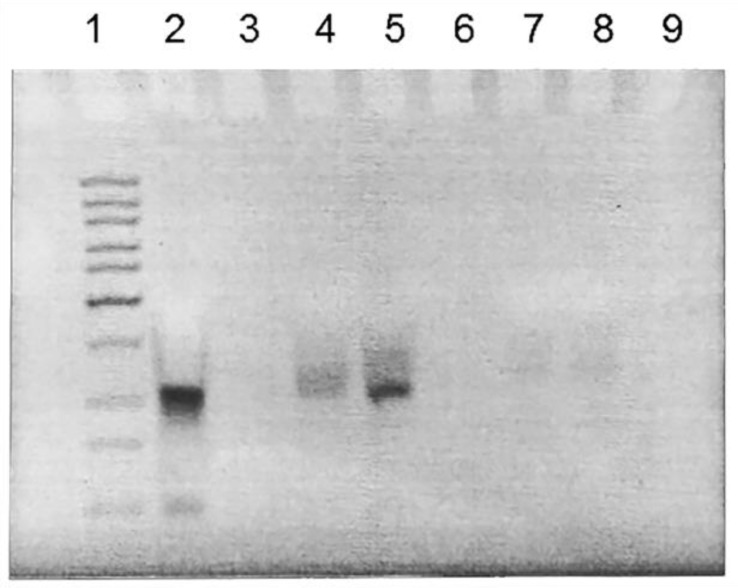
Gel electrophoresis of CPP-AuNP complexes with different oligonucleotide/gold weight-to-weight ratios: 1–10 bp DNA ladder; 2–free oligonucleotide; 3–empty lane; 4–1/10; 5–1/20; 6–1/30; 7–1/40; 8–1/80; 9–empty lane.

The stability of CPP-AuNP complexes with nucleic acids was studied by electrophoresis in 15% polyacrylamide gel (Figure 2). We investigated complexes with different oligonucleotide/gold weight-to-weight ratios, namely 1/10, 1/20, 1/30, 1/40, and 1/80. Note that we used model oligonucleotides in these experiments instead of siRNA. Taking the absence of visible unbound nucleic acid in the gel as a criterion for complex stability, the best stability was demonstrated by CPP-AuNP complexes corresponding to 1/40 oligonucleotide/gold ratios.

### 2.3. Investigation of the Influence of CPP-AuNPs on SEAP Secretion

**Figure 3:**
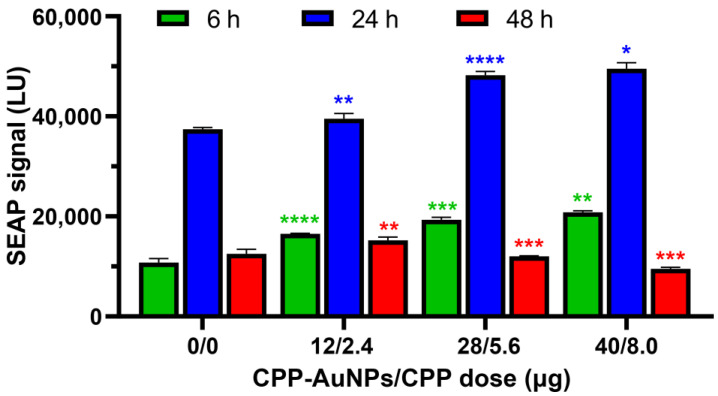
SEAP levels in the SEAP-ARPE-19 cell supernatants (mean ± SD, n = 5) over different time periods after transfection with CPP-AuNPs in Opti-MEM. For statistical analysis, see Table S1. The statistical significance are denoted by: *—*p* ≤ 0.05; **—*p* ≤ 0.01; ***—*p* ≤ 0.001; ****—*p* ≤ 0.0001.

The next series of experiments was devoted to the investigation of the influence of CPP-AuNPs on SEAP secretion: it was demonstrated that the use of developed CPP-AuNPs increased SEAP secretion (Figure 3). The increase appeared to be dose-dependent and exhibited a near-linear trend at 6 h and 24 h after transfection; however, the effect diminished and even partially reversed at 48 h.

### 2.4. Gene Silencing

**Figure 4:**
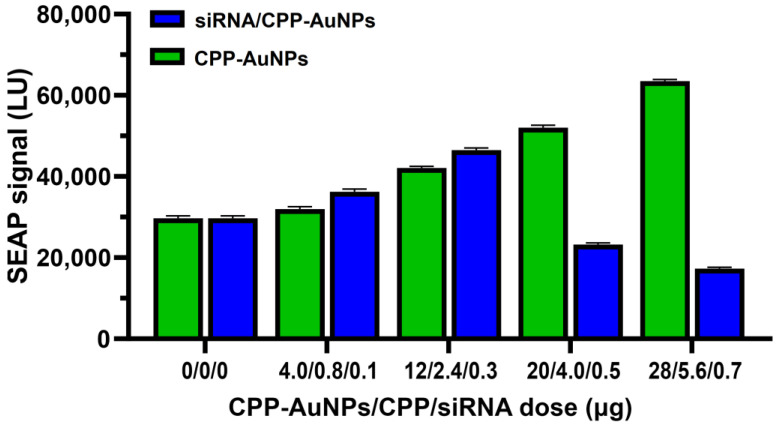
The mean SEAP secretion levels (±SD, n = 7) in SEAP-ARPE-19 cells, measured 24 h after treatment with siRNA/CPP-AuNPs and CPP-AuNPs. For statistical analysis, see Table S2.

The sequence 1795 (5′-UGA CAA CGG GCA ACA ACU CdTdT-3′) was found to be effective at a dose of 0.5 μg when complexed with CPP-AuNPs (Figure 4) but showed no activity when used as naked siRNA.

### 2.5. Cell Viability

Table 1 shows the amounts of siRNA, CPP-AuNP, and CPP used in typical transfections, as well as the toxicity of CPP at various concentrations determined with the MTT assay.

First, we tested the toxicity of CPP from 2 to 45 μM (0.6−13.2 μg), performing the assay 24 h after transfection in a serum-free medium. We found that the peptide was non-cytotoxic up to 15 μM (4.4 μg per well) and exhibited 50% cytotoxicity at 30 μM (8.8 μg per well).

**Figure 5:**
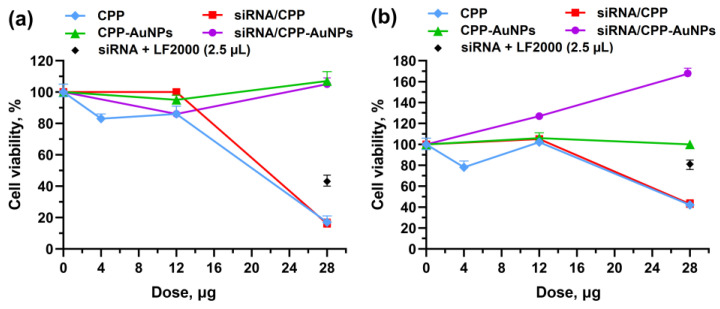
Cell viability (% of that of untreated cells) 48 h after treatment with CPP, CPP-AuNP, siRNA/CPP, siRNA/CPP-AuNP, and siRNA/LF 2000 in: (**a**) serum-free medium (Opti-MEM) and (**b**) serum-containing medium (DMEM/F-12). The doses indicate: for CPP treatment—added CPP mass, in other cases—added AuNPs mass. For statistical analysis, see Tables S3 and S4.

Naked siRNA did not show any significant cytotoxicity. The 48-h cytotoxicity levels of 60% and 22% were observed under using Lipofectamine 2000 for transfection of 0.7 μg of siRNA (i.e., the active siRNA concentration), 2.5 μL per well, when the transfection was performed in a serum-free medium and a serum-containing medium, respectively. The results of cell viability testing 48 h post-transfection in a serum-free medium (Opti-MEM) and in a serum-containing medium (DMEM/F-12) are presented in Figure 5a,b, correspondingly. Neither CPP-AuNPs nor siRNA/CPP-AuNPs samples showed elevated cytotoxicity.

## 3. Discussion

During the development of the synthetic procedure of peptide-modified AuNPs, one of the most important parameters to optimize was the ratio between cysteamine and CPP in order to adjust the attachment of two to three CPP molecules per nanoparticle. Stable complexes can be obtained at a molar ratio of cysteamine to cell-penetrating peptides, corresponding to 40/1. The TEM images showed gold nanospheres with diameters of 22.1, 20.9, and 13.4 in three different batches with corresponding SDs of 4.5, 3.5, and 2.0 nm. The UV–Vis spectra of CPP-AuNPs showed a localized surface plasmon resonance band at 520 nm, characteristic of AuNPs (Figure 1b). The absorbance of siRNA/CPP-AuNP complexes shifted towards longer wavelengths and broadened. That might reflect some degree of interparticle self-assembly due to the presence of both negative and positive charges.

Another parameter to adjust was the optimal ratio between siRNA:CPP-AuNPs in their complexes. The most stable complexes were found to have a 1:40 ratio of the oligonucleotide to gold (*w*/*w*), as indicated by the lack of free oligonucleotide seen in gel electrophoresis (Figure 2). These complexes were selected for use in biological experiments. Note that previous studies have demonstrated that a mass ratio of DNA to cysteamine-coated AuNP complexes, corresponding to 1 to 17, is sufficient for efficient cell transfection using plasmid DNA as an example [64].

In the first SEAP production assay, the treatment of cells with CPP-AuNPs resulted in an increase in SEAP secretion compared to untreated cells (Figure 3). The observed increase in SEAP secretion appeared to be dose-dependent and exhibited a close-to-linear relationship at both 6 and 24 h post-transfection; however, this effect was lost and even slightly reversed at 48 h. The origin of the observed phenomenon is not entirely clear. One of the possible reasons is that siRNA starts degrading at 48 h and thereby loses proper functionality. Additionally, our investigations demonstrated an increase in the viability of cells when incubated with siRNA/CPP-AuNPs. The effect may be attributed to a slight reduction in viability of the engineered SEAP-ARPE-19 cells compared to that of wild-type ARPE-19 cells due to the introduction of a foreign gene, followed by a partial recovery of the viability because of gene silencing. The first transfections were performed using the previously reported anti-SEAP siRNA-2217 (antisense strand: 5′-ACT CTC TGA CAT ACA TCA C-3′) [65] but no effect was observed for doses below 0.7 μg.

The sequence 1795 (5′-UGA CAA CGG GCA ACA ACU CdTdT-3′) exhibited activity when combined with CPP-AuNPs at a concentration of 0.5 μg (as shown in Figure 4) but was ineffective in its naked form as siRNA. At a dose of 0.7 μg, it resulted in a 49% reduction in gene expression compared to untreated cells. The gene silencing effect was even more pronounced (75% reduction) if compared with the increased SEAP levels induced by CPP-AuNPs.

We also tested cell viability in the conditions used for their transfection in our experiments. Importantly, it has been previously shown using various cell lines that the Tat peptide does not exhibit toxic activity, at least at concentrations up to 100 μM [66]. The sequence used in this study was previously shown to be non-toxic to the ARPE-19 cell line at a concentration of 2 μM [67]. 

Here, the concentration of CPP might be even lower if only the peptide bound to AuNPs was considered. Nevertheless, it is important to note that the solution may still contain free, unbound peptides even after dialysis. Therefore, we tested the cell viability using the maximum estimated concentration based on the amount of peptide added during synthesis, which was 0.16 mg/mL. First, we assessed the cytotoxicity of CPP in the range from 2 to 45 μM (0.6−13.2 μg), performing the assay 24 h post-transfection in a serum-free medium. Interestingly, an LD50 corresponding to a concentration of 30 µM was demonstrated, while a concentration of 15 µM was found to be practically non-toxic.

However, results from the MTT assay conducted 48 h after transfection showed significantly lower levels of cytotoxicity for the peptide (Table 1); it did not show 90% toxicity until 95 μM (28 μg), whereas in the assay after 24 h it reached the same level of cytotoxicity at 44 μM (13 μg). Hypothetically, this can be explained by the initial post-transfection shock, followed by gradual proliferation recovery. After repeated 48-hour MTT assays, it is clear that significant toxicity starts at 95 μM (28 μg), which is beyond the maximal theoretic CPP concentration in the CPP-AuNP solution. The cytotoxicity due to the peptide presence should thus not be an issue. In addition, naked siRNA did not show any significant cytotoxicity. Thus, we have to assume that the cytotoxicity of CPP or CPP-AuNPs, and their siRNA complexes cannot be a problem in the application of the reported AuNPs for oligonucleotide intracellular delivery. The reduced cytotoxicity of the AuNP formulations may be explained by the masking effect of gold nanoparticles on the toxic properties of naked CPP, thus it generates different mechanisms of interaction with the cell membrane. Moreover, to minimize AuNPs cytotoxicity, assembling such agents from hybrid Au/Fe_3_O_4_ nanostructures could be a possible solution [39].

Meade and Dowdy et al. have argued that non-covalent siRNA/CPP complexes are the best approach, since the positive charge of the CPP may be neutralized by covalently attached siRNA [68]. Furthermore, they state that the enhanced delivery of covalent conjugates may be in fact due to the excess of free peptides in the mixture. As in our case, there were nearly no free peptides in the mixture due to the purification procedure (dialysis), we are unable to accept such an explanation. We assume that the enhanced delivery of siRNA was induced by its complex formation with positively charged AuNPs, and the cell permeability of the complex was mediated by CPPs covalently attached to those AuNPs.

Notably, the functionalization principle of the developed CPP-AuNP with nucleic acids is primarily based on electrostatic interactions. This makes the principle quite versatile for the creation of agents of other types, for example, plasmid-functionalized vectors. However, the development of each novel agent will require optimization of parameters, e.g., empirical identification of the proper ratio of the particle/plasmid amounts, along with the experimental verification of their operation.

Future prospects of the present research include extensive studies of the intracellular fate of silencing agents obtained according to the proposed method. At this point, the observed gene silencing mediated by the developed siRNA/CPP-AuNPs suggests that at least some of the agents escape from endosomes. Meanwhile, many research papers, which address the post-uptake intracellular localization of functionalized AuNPs, demonstrate the influence of a multitude of factors, such as coating, size, shape, potential, and many others, on the particle fate after delivery, and no universal scheme was revealed [69,70,71]. Therefore, such aspects as post-delivery pathways of the particles and the intactness of the delivered oligonucleotide require thorough investigation, particularly with regard to enhancing the silencing efficiency by affecting the post-delivery fate.

As the present research does not include a profound examination of the intracellular fate of the developed siRNA/CPP-AuNPs complexes, there is a possibility that they may be taken up by lysosomes, where they could be either degraded or directed to other organelles. Such events may cause further disruptions in the cellular redox system and potentially result in alterations in the intracellular pH.

The findings of the present research can be used for the development of methods for siRNA delivery in vitro. Although the CPP-AuNP-based delivery methods have the potential to provide valuable insights for novel therapeutic approaches [72,73,74], it is important to note that their application will require a separate comprehensive investigation of in vivo immunogenicity and off-target effects, along with optimization of all parameters in each case.

## 4. Materials and Methods

### 4.1. Peptide Synthesis

The Tat-linked CPP (H-CGRKKRWWPQRWWRWWRPPQ-OH) was generated based on the methods described in Ref. [67] using an ACT-396 peptide synthesizer and following the Fmoc-chemistry protocols using Wang resin. The coupling reagent employed was TBTU/HOBT, while Fmoc deprotection was performed using 2% DBU/2% piperidine in NMP. For final cleavage and deprotection, a blend of phenol, H_2_O, EDT, thioanisol, and TFA was applied to the resin at room temperature for 2 h in the ratio of 1.5:1:1:1:10. Coupling reagents, derivatives of amino acids, and resins used in this work were procured from GL Biochem Ltd., Shanghai, China. The crude peptide was precipitated from the cleavage mix with ice-cold diethyl ether, filtered, and dried. Purification was achieved through reverse phase-high performance liquid chromatography (RP-HPLC) using a Supelco AscenticTM C18 semi-preparative column with dimensions of 10 × 150 mm and particle size of 5 μm. HPLC traces of crude peptide as well as the profile of elution of peptide after purification were very similar to those published in supplementary materials of Ref. [67]. After the purification, the peptide was lyophilized and stored at −20 °C.

### 4.2. Oligonucleotide Synthesis

All siRNAs, including the model double-stranded oligodeoxyribonucleotide, were synthesized using an ASM-800 DNA/RNA synthesizer (Biosset, Novosibirsk, Russia). Each strand was synthesized separately by following the standard RNA synthesis procedures and utilizing TOM-protected monomers (Glen Research, Sterling, VA, USA). The cleavage and deprotection protocols specified by the supplier were followed. Purification of synthesized oligonucleotides was performed by IE HPLC followed by desalting by PolyPak II cartridges (Glen Research) according to standard protocols. The siRNA concentration was determined by measuring the absorbance of each strand at 260 nm and using conversion factor of 1 OD being equal to 40 μg/mL of single-stranded RNA. The siRNA strands were immediately annealed before conducting biological experiments, using Dharmacon siRNA buffer consisting of 20 mM KCl, 6 mM HEPES, 0.2 mM MgCl_2_ × 6H_2_O, and a pH of 7.5.

### 4.3. Synthesis of CPP-AuNPs

The cell-penetrating peptide-modified gold nanoparticles (CPP-AuNPs), with a 40:1 (mol/mol) ratio of the surface stabilizing agent (cysteamine) to CPP, were prepared as follows: 5 mg (14 μmol) of gold (III) chloride trihydrate (ACS Reagent, Sigma-Aldrich, St. Louis, MO, USA) was dissolved in 5 mL of deionized water. After that, a solution containing 1.55 mg (0.53 μmol) of the peptide and 2.4 mg of cysteamine in 4 mL of dH_2_O was added while stirring at room temperature and incubated for 5 min. A solution containing 0.02 mg of NaBH4 (Sigma-Aldrich, ≥98.5%) in 0.5 mL of deionized water was added dropwise over a 20-minute period. The mixture was then stirred vigorously and left overnight. The mixture was then moved to a dialysis tube (Medicell International Ltd., London, UK, MWCO 12–14 kDa) and dialyzed against dH_2_O for 48 h. The final product, a suspension of CPP-AuNPs, was stored in a glass container at room temperature. Additional CPP-AuNPs were produced using the same method, with only the ratio between the CPP and cysteamine being altered. The CPP-AuNPs concentration was estimated by assuming complete reduction of gold chloride present in the reaction mixture. This calculation was based on the amount of gold chloride used, which was 5 mg, and the total volume of the reaction solution, which was 9.5 mL. This resulted in a CPP-AuNPs concentration of 0.5 mg/mL gold. The data on the size of the particles (measured to be 20 nm) was utilized to calculate the number of gold atoms per particle and the molar concentration of the solution through application of equations from Ref. [75].

### 4.4. Preparation of siRNA-AuNP Complexes

The siRNA-AuNP complexes were prepared by adding double-stranded siRNA in RNase-free water to CPP-AuNP in different equivalents, and their stability was evaluated using electrophoresis with a double-stranded oligodeoxyribonucleotide as an siRNA model (as described in the subsequent section).

### 4.5. Characterization of AuNP and siRNA/AuNP Complexes

CPP-AuNPs were characterized with JEOL 1200-EX II transmission electron microscope (JEOL, Tokyo, Japan), on carbon-coated copper grids, with 100–200 k magnifications. The particle diameters were measured manually using Scion Image 4.0.2 software (Scion Corporation, Chicago, IL, USA).

The optical properties of both the CPP-AuNPs and siRNA/CPP-AuNP complexes were measured using absorption spectroscopy, covering a wavelength range from 200 to 800 nm. All the measurements were performed using a Cary 100 UV–Vis spectrophotometer (Varian, Palo Alto, CA, USA).

The presence of free CPP in the reaction mixture was assessed through RP-HPLC analysis after the completion of CPP-modified AuNP formation. The CPP-AuNPs were precipitated by adding 100 μL of 2 M HCl and then centrifuged. The amount of unattached peptide was analyzed by RP-HPLC, following the same procedure for peptide synthesis, and compared to a calibration curve generated for peptide concentrations ranging from 0.05 to 1.0 mg/mL.

A synthetic double-stranded oligodeoxynucleotide, which possessed the identical sequence as the functional siRNA, was used as a representative molecule to evaluate the stability of the siRNA/CPP-AuNP complexes. The complex stability at different oligonucleotide:peptide mass ratios was tested with 15% PAGE. Stable complexes could be indicated by the lack of unbound oligonucleotide in the electrophoresis gel.

### 4.6. SEAP-ARPE-19 Cells Engineering

Human ARPE-19 retinal pigment epithelial cells were incubated overnight in 100 mm dishes (initial concentration—1 million cells per dish). Then, the cells were exposed to a mixture of 7 μg pCMV-SEAP2/neo reporter plasmid and 25 kDa branched polyethyleneimine (PEI25) with a charge ratio of +4. G418 was added to culture medium at a concentration of 0.8 mg/mL as a selective agent. After the colonies formed, they were harvested and grown in 48 wells plated with medium containing 0.6 mg/mL G418. The expression of alkaline phosphatase was determined through a chemiluminescent assay (The Great EscAPe SEAP Kit, Clontech, Mountain View, CA, USA) and multiple colonies exhibiting varying levels of expression and normal growth were chosen for further experiments.

### 4.7. Transfection of SEAP-ARPE-19 with CPP-GNPs

Engineered SEAP-ARPE-19 cells were cultured in nutrient-rich media consisting of DMEM/F-12, fetal bovine serum, L-glutamine, penicillin, streptomycin, and G418. Incubation was performed at a temperature of 37 °C with 7% CO_2_. To maintain growth, the cells were subcultured weekly.

For further transfection, the cells were seeded in 96-well plates and grown in 100 μL of growth medium. After 24 h, the transfection mixtures were added and the cells were either washed with PBS or grown in fresh medium. The impact of CPP-AuNPs on SEAP expression was monitored over time at 6, 24, and 48 h post-transfection, and the effect on cell viability was studied 24 or 48 h post-transfection. Cell proliferation was also studied by counting the number of transfected and non-transfected cells at 24 and 48 h post-transfection using erythrosine dye and a manual cell counter.

### 4.8. SEAP Production Analysis

Great EscAPe Chemiluminescence Kit 2.0 (Clontech) was used to measure the SEAP levels in cell supernatants. The assay was performed according to the protocol recommended by the manufacturer. Briefly, a 60-microliter aliquot was taken from each sample of culture medium. The samples were centrifuged at 12,000 rpm for 1 min and the supernatants were frozen at −20 °C for future analysis. Before assaying, samples were equilibrated to room temperature and then were diluted 20-fold in conditioned growth medium and 15 μL of each was transferred to a 96-well plate (white IsoPlate/OptiPlate, PerkinElmer, Waltham, MA, USA). Then, 45 μL of the kit 1× Dilution Buffer was added to each sample. The plate was sealed and incubated at 65 °C for 30 min, after that samples were cooled on ice for 2–3 min and equilibrated to room temperature. After that, the samples were incubated for 60 min with SEAP substrate solution (60 μL). Finally, the SEAP signal was detected and recorded using either a Microbeta 1450 luminometer/liquid scintillation counter (Wallac) or VarioSkan Flash multimode reader (Thermo Scientific, Waltham, MA, USA).

### 4.9. MTT Assay

MTT assay was used to investigate the impact of transfection on cell viability. Solution of MTT in PBS (5 mg/mL) was previously prepared and filtered with a 0.2 μm filter. After transfection and following incubation (during 24 or 48 h), cells were rinsed with PBS and a mixture of 10 parts of DMEM/F-12 and 1 part of the MTT solution was added to each well, including control wells without cells and wells with non-transfected cells. Incubation was performed for 2 h at a temperature of 37 °C with 7% CO_2_. Then, the medium was removed and 100 μL of DMSO was added to each well to dissolve the formazan crystals produced by living cells. VarioSkan Flash multimode reader was used for absorbance measurements at a wavelength of 570 nm. To put in evidence that AuNPs did not affect the MTT reading, cells transfected with high concentrations of CPP-AuNPs were tested via modified assay procedure where only culture medium was added to cells instead of the described mixture.

### 4.10. Statistical Analysis

The results of each group are expressed in mean ± standard deviation (SD; n = 5 for Figure 3 and Figure 5 and n = 7 for Figure 4), and all the experimental values were compared with the corresponding control values. The results were presented as mean ± standard deviation and were analyzed in GraphPad Prism version 8 (GraphPad Software Inc., La Jolla, CA, USA) using multiple *t*-tests to determine whether the values were to be considered statistically significant (*p* value ≤ 0.05). The following marks were used to reflect statistical significance: ns—*p* > 0.05; *—*p* ≤ 0.05; **—*p* ≤ 0.01; ***—*p* ≤ 0.001; ****—*p* ≤ 0.0001.

## 5. Conclusions

A straightforward method to synthesize positively charged AuNPs with surface modification by cell-penetrating peptides, and without any additional steps of polymer surface modifications, has been developed. The synthesized peptide-modified AuNPs were thoroughly characterized by the TEM and SPR techniques and demonstrated a good ability to complex molecules of nucleic acid nature as well as a capacity to serve as vehicles for gene therapy via intracellular delivery without loss of siRNA biological activity. Importantly, the obtained nanoconjugates demonstrated low cytotoxicity. In conclusion, the method has the potential to be extended for the creation of novel sensing surfaces [76] and functionalized nanoparticles [77,78], as well as for the targeted delivery of other nanoagents such as those decorated by short non-complementary DNA [79], integrin-binding peptides [80], etc. The developed hybrid nanoagents can be useful tools for the delivery of negatively charged biomolecules such as antisense oligonucleotides, RNAs, etc. for various life science applications.

## Figures and Tables

**Table 1:** The amounts of compounds used for transfections in a serum-free medium (Opti-MEM) and associated cytotoxicity.

siRNA (µg)	CPP-GNP (µg)	CPP, Max. (µg/µM)	CPP Cytotoxicity (24 h)	CPP Cytotoxicity (48 h)
0.1	4	1.3/4.4	Non-cytotoxic	Non-cytotoxic
0.3	12	3.8/13	Non-cytotoxic	12–24%
0.7	28	9/31	50%	n/d *
1.0	40	12.8/44	90%	11–29%
0	0	28/95	n/d *	55–88%
0	0	45/153	n/d *	98%

* n/d stands for “not determined”.

## Data Availability

The data presented in this study are available in this article and its supplementary materials.

## Supplementary Materials

The following supporting information can be downloaded at: https://www.mdpi.com/article/10.3390/molecules28083318/s1, Table S1: Statistical analysis for the data presented in Figure 3. SEAP production by SEAP-ARPE-19 cells 6 h, 24 h, and 48 h post-transfection with CPP-GNPs in Opti-MEM; Table S2: Statistical analysis for the data presented in Figure 4. SEAP secretion by SEAP-ARPE-19 cells 24 h post-transfection; Table S3: Statistical analysis for the data presented in Figure 5a. Cell viability 48 h post-transfection in serum-free medium Opti-MEM; Table S4: Statistical analysis for the data presented in Figure 5b. Cell viability 48 h post-transfection in serum-containing medium DMEM/F-12.

## Sample Availability

Samples of the compounds are not available from the authors.
