# Supplementary material for: A Straightforward Method for the Development of Positively Charged Gold Nanoparticle-Based Vectors for Effective siRNA Delivery
Source: Molecules. 2023 Apr 8;28(8):3318. doi: 10.3390/molecules28083318 (PMC10144622; doi:10.3390/molecules28083318)
Supplement: Supplementary file 1 [file molecules-28-03318-s001.zip › molecules-2296497-supplementary.pdf]

Supplementary Information

A Straightforward Method for the Development of Positively Charged Gold Nanoparticle-Based Vectors for Effective siRNA Delivery

Tatiana N. Elizarova [1], Maxim L. Antopolsky [1], Denis O. Novichikhin [1,2], Artemiy M. Skirda [1,2], Alexey V. Orlov [1,*], Vera A. Bragina [1] and Petr I. Nikitin [1,2,*]

[1] Prokhorov General Physics Institute, Russian Academy of Sciences, 119991 Moscow, Russia
[2] National Research Nuclear University MEPhI (Moscow Engineering Moscow Engineering Physics Institute (MEPI), National Research Nuclear University, 115409 Moscow, Russia
[*] Correspondence: alexey.orlov@kapella.gpi.ru (A.V.O.); nikitin@kapella.gpi.ru (P.I.N.)

Table of content:

Table S1. Statistical analysis for the data presented in Figure 3........2

Table S2. Statistical analysis for the data presented in Figure 4........2

Table S3. Statistical analysis for the data presented in Figure 5a........2

Table S4. Statistical analysis for the data presented in Figure 5b........3

Table S1. Statistical analysis for the data presented in Figure 3. SEAP production by SEAP-ARPE-19 cells 6 h, 24 h and 48 h post-transfection with CPP-AuNPs in Opti-MEM

Time of SEAP measurement	Dose of CPP/AuNPs added (μg)	Number of measurements	Mean (LU)	Upper limit (LU)	Lower limit (LU)
6 h	0	5	10750	800	100
6 h	12	5	16500	100	50
6h	28	5	19300	500	100
6 h	40	5	20800	300	100
24 h	0	5	37400	350	150
24 h	12	5	39500	1050	200
24 h	28	5	48200	750	200
24 h	40	5	49500	1200	150
48 h	0	5	12500	900	100
48 h	12	5	15200	650	50
48 h	28	5	12000	100	100
48 h	40	5	9500	300	200

Table S2. Statistical analysis for the data presented in Figure 4. SEAP secretion by SEAP-ARPE-19 cells 24 h post-transfection

Dose of siRNA/CPP-AuNPs complex added (μg)	Number of measurements	Mean (LU)	Upper limit (LU)	Lower limit (LU)
0/0	7	29700	600	100
0.1/4	7	36200	700	200
0.3/12	7	46500	500	100
0.5/20	7	23200	400	200
0.7/28	7	17300	300	100

Table S3. Statistical analysis for the data presented in Figure 5a. Cell viability 48 h post-transfection in serum-free medium Opti-MEM.

Compounds added	Dose, μg	Number of measurments	Mean (%)	Upper limit (%)	Lower limit (%)
CPP	4	5	83	3	0.5
CPP	12	5	86	5	1
CPP	28	5	17	4	1
CPP-AuNP	12	5	95	3	1
CPP-AuNP	28	5	107	6	2
siRNA/CPP	12	5	100	2	1
siRNA/CPP	28	5	16	3	1
siRNA/CPP-AuNP	12	5	86	5	2
siRNA/CPP-AuNP	28	5	105	4	1

Table S4. Statistical analysis for the data presented in Figure 5b. Cell viability 48 h post-transfection in serum-containing medium DMEM/F-12.

Compounds added	Eq. dose of CPP, μg	Number of measurments	Mean (%)	Upper limit (%)	Lower limit (%)
CPP	4	5	78	6	2
CPP	12	5	102	4	1
CPP	28	5	42	3	1
CPP-AuNP	12	5	106	5	2
CPP-AuNP	28	5	100	3	0
siRNA/CPP	12	5	105	3	1
siRNA/CPP	28	5	43	4	2
siRNA/CPP-AuNP	12	5	127	3	2
siRNA/CPP-AuNP	28	5	168	5	1
